# Nucleolar Proteomics Revealed the Regulation of RNA Exosome Localization by MTR4

**DOI:** 10.1016/j.mcpro.2025.101031

**Published:** 2025-07-10

**Authors:** Yaqian Zhang, Guangzhen Jiang, Ke Wang, Minjie Hong, Xinya Huang, Xiangyang Chen, Xuezhu Feng, Shouhong Guang

**Affiliations:** 1 Department of Obstetrics and Gynecology, The First Affiliated Hospital of USTC, The USTC RNA Institute, Ministry of Education Key Laboratory for Membraneless Organelles & Cellular Dynamics, Hefei National Research Center for Physical Sciences at the Microscale, Center for Advanced Interdisciplinary Science and Biomedicine of IHM, School of Life Sciences, Division of Life Sciences and Medicine, University of Science and Technology of China, Hefei, Anhui, China; 2 Guangzhou National Laboratory, Bio-Island, Guangzhou, Guangdong, China; 3 School of Basic Medical Sciences, Anhui Medical University, Hefei, China

**Keywords:** proteomics, RNA exosome, MTR4, translocation, nucleolus

## Abstract

The nucleolus is the largest membrane-less organelle within the nucleus and plays critical roles in regulating the cell cycle, senescence, and stress responses. The RNA exosome is a multiprotein ribonucleolytic complex involved in RNA processing and degradation in the cytoplasm, the nucleus, and the nucleolus. Previous studies have shown that the subcellular localization of the RNA exosome is crucial for its function. However, the mechanism that regulates its spatial distribution remains largely unexplored. In this study, we identified the nuclear RNA helicase MTR4 as a regulator of the RNA exosome localization through nucleolar quantitative proteomics technology. Immunostaining and fluorescence tagging confirmed that the depletion of MTR4 resulted in the translocation of the RNA exosome subunits from the nucleolus to the nucleoplasm. Notably, the translocation is specifically regulated by MTR4 and does not depend on other cofactors of the MTR4-containing Trf4/5-Air1/2-Mtr4 polyadenylation, poly(A) exosome-targeting, and nuclear exosome targeting complexes. The nucleolar accumulation of exosome subunits mutually depends on other exosome subunits. Additionally, actinomycin D treatment, which inhibits transcription, induced the RNA exosome to translocate from the nucleolus to the nucleoplasm, likely through the regulation of nucleolar MTR4 levels. These findings uncover a regulatory mechanism that modulates the localization of the RNA exosome within the nucleolus.

## Highlights

• Quantitative nucleolar proteomics reveals that MTR4 modulates the nucleolar proteome.
• The depletion of MTR4 reduces the nucleolar enrichment of the RNA exosome.
• The transcription process promotes the nucleolar accumulation of the RNA exosome.
• The integrity of the RNA exosome is essential for its nucleolar localization.

## Graphical Abstract

**Figure:**
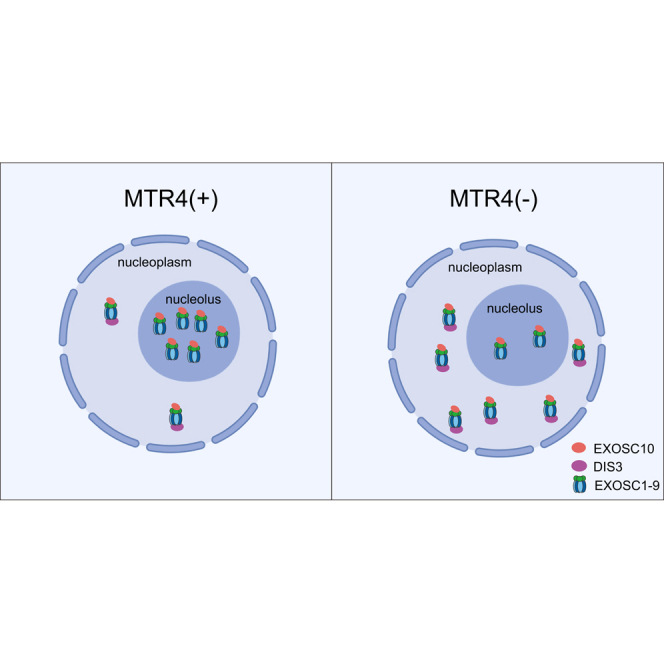

## In Brief

Biochemical fractionation followed by proteomics and fluorescent imaging approaches revealed the RNA helicase MTR4-modulated nucleolar proteome. The depletion of MTR4 or inhibiting transcription both redistributed the RNA exosome complex from the nucleolus to the nucleoplasm.

The nucleolus is a central organelle responsible for coordinating the synthesis and assembly of ribosomal subunits, forming around clusters of repeated ribosomal genes (1). Previous reports have suggested that the nucleolus of mammalian cells consists of four internally phase-separated subcompartments: the fibrillar center (FC), the dense fibrillar component (DFC), the granular component (GC), and a newly identified periphery of the dense fibrillar component (PDFC) (2).

The FC is the subcellular compartment where ribosomal transcription occurs. Each FC contains two to three transcriptionally active ribosomal DNA (rDNA) units and is encircled by pre-rRNA processing factors, which are then assembled into the DFC (3). Nascent rRNA transcripts undergo sequential processing steps by enzymes that localize to distinct intranucleolar subcompartments. Rapid rRNA transcription occurs at the boundary between the FC and the DFC, and pre-rRNA processing takes place within the DFC. The PDFC, located on the outermost side of the DFC, contains some proteins that facilitate the removal of the 3′ external transcribed spacer (3′ ETS) (4). Each nucleolus houses multiple FC/DFC units within a single GC region, where the final stages of pre-rRNA processing and the assembly of rRNA ribonucleoproteins take place (5).

In addition to mediating rRNA transcription and processing, the nucleolus functions as a non-membrane-bound nuclear organelle that controls protein quality. The nucleolar proteome is rich in stress-sensitive proteins, such as the RNA exosome and the heat shock proteins (HSPs). The nucleolus temporarily stores misfolded proteins to prevent their irreversible aggregation, facilitates Hsp70-assisted refolding, and promotes nuclear protein homeostasis under stress (6).

The nucleolar proteome is dynamic, characterized by disassembly and reassembly under various cellular conditions and throughout different phases of the cell cycle, reflecting its intrinsic mobility (1, 7, 8). Previous analyses identified over 4500 proteins in the nucleolus across various cell lines, most of which are involved in ribosome biogenesis, including rRNA transcription, processing, and ribosome assembly (9, 10, 11). Proteomic studies have revealed complex reorganizations of the nucleolar proteome during stress responses, such as the inhibition of transcription, viral infection, heat shock, and DNA damage (1, 12, 13, 14, 15). Furthermore, spatial proteomics facilitates proteome-wide comparisons of proteins’ subcellular localization changes in response to various perturbations (13).

The RNA exosome is an evolutionarily conserved ribonucleolytic complex composed of ten or eleven subunits. It plays a central role in the processing and maturation of various RNAs, as well as in the degradation and surveillance of aberrant RNAs, thereby maintaining RNA homeostasis (16, 17, 18, 19). In budding yeast, the nine-subunit exosome core (Exo9) exhibits distinct subcellular localization patterns: it associates with Rrp44 to form the cytoplasmic Exo10 complex, while combining with Rrp6 to constitute the nuclear Exo11 complex. These differentially localized complexes interact with a conserved eukaryotic RNA processing complex containing specific cofactors and RNA substrates according to their cellular compartmentalization (17, 20, 21). Notably, all exosome subunits show predominant nucleolar localization, consistent with their essential roles in nuclear RNA processing (21, 22). In human cells, six RNA exosome subunits (EXOSC4–EXOSC9) form a barrel-like hexameric PH ring that serves as a scaffold for RNA substrates. Three subunits EXOSC1–EXOSC3 constitute an S1-KH cap, while EXOSC10 and DIS3 function as the two catalytic subunits (23, 24). EXOSC1, also known as Rrp41 or Csl4, is one of the core components of the RNA exosome complex. EXOSC5, also known as Rrp46, is a non-catalytic subunit that helps recruit the RNA exosome complex to target RNAs, facilitating their degradation. The coordinated activities of EXOSC1 and EXOSC5 are essential for maintaining the functional integrity of the RNA exosome, enabling efficient RNA metabolism and quality control (25). EXOSC10, also known as Rrp6, is predominantly enriched in the nucleolus and serves as the crucial catalytic subunit of the RNA exosome (26).

Accessory factors of the RNA exosome complex determine its RNA substrate specificity (27). The nuclear exosome targeting (NEXT) complex consists of MTR4 (a helicase of the SKI2-like family), the zinc-finger–containing protein ZCCHC8, and the RNA-binding protein RBM7. This complex primarily targets short, mono-exonic RNAs, including promoter upstream transcripts/upstream antisense RNAs, enhancer RNAs, and long noncoding RNAs (28). The poly(A) exosome-targeting (PAXT) complex, composed of MTR4 and the zinc-finger protein ZFC3H1, directs nuclear noncoding transcripts with longer poly(A) tails to the RNA exosome for degradation (29). The Trf4/5-Air1/2-Mtr4 polyadenylation (TRAMP) complex consists of MTR4, a poly(A) polymerase (PAPD5), and a zinc knuckle protein (ZCCHC7). It mediates the degradation of aberrant pre-rRNA intermediates, defective pre-rRNAs, and the 5′ ETS (30, 31, 32). MTR4, a conserved 3′-5′ DExH-box RNA helicase, plays a key role in unwinding and degrading structured RNA substrates (33). All these complexes include MTR4, which also participates in RNA export from the nucleus to the cytoplasm (34). Moreover, MTR4 monitors the quality of nuclear RNA before export, ensuring that only properly processed RNAs are transported to the cytoplasm (35, 36). MTR4-dependent RNA surveillance serves as a critical checkpoint for oocyte growth (37).

Our previous work revealed that the RNA exosome can translocate from the nucleolus to the nucleoplasm upon cold-warm exposure and subsequently restores its nucleolar accumulation during recovery in *Caenorhabditis elegans*. Additionally, in suppressor of siRNA (*susi*) mutants, where erroneous rRNAs accumulate, the RNA exosome mislocalizes from the nucleolus to the nucleoplasm (38, 39). Notably, EXOSC10 can relocate from the nucleolus to DNA damage sites to facilitate efficient homologous recombination (40, 41, 42). These findings collectively suggest that distinct subcellular localizations may provide specialized environments and interaction partners for the RNA exosome to perform its functions (19, 43). Understanding the regulatory mechanisms of RNA exosome subcellular localization is essential for elucidating its functions.

Here, by isolating nucleoli followed by mass spectrometry analysis, we showed that the knockdown of MTR4 induced the translocation of the RNA exosome from the nucleolus to the nucleoplasm. MTR4 specifically regulates the nucleolar localization of the RNA exosome, without disrupting nucleolar integrity. Additionally, knocking down subunits of the RNA exosome complex mutually affected the localization of other subunits. Treatment with a series of chemicals, including actinomycin D (Act.D), MG-132, and leptomycin B (LMB), altered the nucleolar localization of EXOSC10. In addition, Act.D treatment decreased the nucleolar accumulation of the RNA exosome by regulating nucleolar MTR4 levels. Our work revealed that the subcellular localization of the RNA exosome complex within the nucleolus can be regulated by MTR4.

## Experimental Procedures

### Cell Culture

HeLa cells and 293T cells were cultured in Dulbecco's modified Eagle medium supplemented with 10% fetal bovine serum (FBS), 1% penicillin/streptomycin, 1% non-essential amino acids, and 1% sodium pyruvate. All cells were maintained in a humidified incubator at 37 °C with 5% CO_2_.

### Plasmid Transfection

One day before transfection, approximately 75,000 cells in 500 μl of growth medium without antibiotics were seeded into each well of 24-well plates to achieve 90 to 95% confluency at the time of transfection. Transfection was conducted using the DNA-Lipofectamine 2000 reagent following the vendor’s protocols. Cells were then incubated in a CO_2_ incubator for another 24 to 48 h. The plasmids used are listed in Supplemental Table S3.

### Small-Interfering RNA

siRNAs were purchased from Horizon (Lafayette, CO). The transfection of siRNAs in mammalian cells was performed using DharmaFECT 1 Transfection Reagent (Horizon, T-2001-03) according to the manufacturer recommended procedure. The knockdown efficiency was analyzed by reverse transcription quantitative polymerase chain reaction (RT-qPCR) at 48 and 72 h posttransfection. Western blotting was performed to analyze the protein levels following siRNA treatments. The siRNA target sequences are listed in Supplemental Table S4.

### RNA Isolation and RT-qPCR

Total RNAs were extracted using TRizol Reagent (Invitrogen) according to the manufacturer’s protocol. For RT-qPCR, complementary DNAs were synthesized from RNA using HiScript III RT SuperMix for qPCR (Vazyme), which includes a random primer/oligo(dT)20VN primer mix for reverse transcription. qPCR was performed using a MyIQ2 real-time PCR system (Bio-Rad) with AceQ SYBR Green Master Mix (Vazyme). The primers used in qRT-PCR are listed in Supplemental Table S5.

### Western Blotting

Cells were harvested and washed twice with PBS and then lysed in RadioImmunoPrecipitation assay lysis buffer. The samples were frozen at −80 °C and subsequently lysed at 95 °C for 10 min in 1 × protein dye (62.5 mM Tris, pH 6.8; 10% glycerol; 2% SDS; 5% β-mercaptoethanol; 0.2% bromophenol blue). Proteins were resolved by SDS-PAGE on gradient gels (10% separation gel, 5% spacer gel) and transferred to a nitrocellulose blotting membrane. After washing with 1 × tris buffered saline with tween-20 (TBST) buffer and blocking with 5% milk TBST, the membrane was incubated overnight at 4 °C with primary antibodies. The membrane was washed three times for 10 min each with 1× TBST buffer and then incubated with secondary antibodies at room temperature for 2 h. The membrane was washed three times for 10 min with 1 × TBST buffer and then visualized. The primary and secondary antibodies used are listed in Supplemental Table S6.

### Chemical Treatment

For Act.D treatment, HeLa cells were exposed to 40 nM Act.D (MCE, HY-17559) for 1 h. Control cells received an equivalent concentration of DMSO. For MG-132 treatment, HeLa cells were incubated for 24 h in medium containing 100 nM MG-132 (MCE, HY-13259). HeLa cells were also treated with 80 nM LMB (Beyotime, S1726) for 24 h, while control cells were treated with ethanol. For carbonyl cyanide-4-(trifluoromethoxy)phenylhydrazone (FCCP) treatment, HeLa cells were incubated for 24 h in medium containing 20 μM FCCP (MCE, HY-100410). For cycloheximide (CHX) treatment, HeLa cells were incubated for 48 h in medium containing 2.8 μM CHX (MCE, HY-12320).

### Fluorescence Recovery After Photobleaching

Fluorescence recovery after photobleaching experiments were conducted using a Zeiss LSM980 laser scanning confocal microscope at room temperature as described previously (44). A region of interest was bleached using 100% laser power for 3 to 4 s, and fluorescence intensities in these regions were collected every 5 s and normalized to the initial intensity before bleaching. For analysis, image intensity was measured by mean values and further analyzed using Origin software (https://www.originlab.com/).

### Immunofluorescence and Confocal Microscopy

Cells seeded on coverslips were fixed with 4% paraformaldehyde for 15 min at room temperature and then washed three times with 1× PBS. The cells were subsequently incubated for 15 min in permeabilization buffer (0.2% Triton X-100 and 2% FBS in FACS buffer). The proteins of interest were labeled with primary antibodies at a 1:500 dilution in permeabilization buffer at 4 °C overnight, followed by three washes with washing buffer. The coverslips were then incubated with secondary antibodies at a 1:1000 dilution in permeabilization buffer for 1 h at room temperature in dark. Finally, the coverslips were washed with washing buffer and counterstained with Hoechst 33342 for 10 min at room temperature in the dark. After washing with washing buffer, images were acquired using a Zeiss LSM980 laser scanning confocal microscope with a 100× oil-immersion objective and a 1024 × 1024 image size.

### Nucleolar Fractionation

Nucleolar fraction preparation was performed as previously described (1).

All solutions were supplemented with a complete protease inhibitor tablet at a final concentration of one tablet per 50 ml. The compositions of the buffers were as follows:

Buffer A: 10 mM Hepes (pH 7.9), 10 mM KCl, 1.5 mM MgCl_2_, 0.5 mM DTT.

S1 solution: 0.25 M sucrose, 10 mM MgCl_2_.

S2 solution: 0.35 M sucrose, 0.5 mM MgCl_2_.

S3 solution: 0.88 M sucrose, 0.5 mM MgCl_2_.

Freshly harvested cells were washed twice with PBS, resuspended in 5 ml of hypotonic buffer A, and incubated on ice for 10 min. The cell suspension was transferred to a pre-cooled 7-ml Dounce homogenizer and dounced 35 times on ice. The homogenized cells were centrifuged at 1000 rpm for 5 min at 4 °C to pellet the nuclei, which were enriched but not yet highly pure.

The nuclear pellet was resuspended in 5 ml of hypotonic buffer A, homogenized with 25 strokes in the Dounce homogenizer, and centrifuged again at 1000 rpm for 5 min at 4 °C. The resulting pellet was resuspended in 3 ml of S1 solution and carefully layered over 3 ml of S2 solution, ensuring the two layers remained cleanly separated. This mixture was centrifuged at 2500×*g* for 5 min at 4 °C, yielding a cleaner nuclear pellet.

The nuclear pellet was resuspended in 3 ml of S2 solution by pipetting up and down, then sonicated using eight 10-s bursts at 40% power, with 10-s intervals between bursts. The sonicated sample was layered over 3 ml of S3 solution and centrifuged at 3500×*g* for 10 min at 4 °C. The supernatant was gently removed, and the nucleoli were resuspended in 0.5 ml of S2 solution, followed by centrifugation at 2500×*g* for 5 min at 4 °C. The final pellet, containing highly purified nucleoli, was resuspended in 0.5 ml of S2 solution and stored at −80 °C.

### LC-MS/MS Analysis

Peptide samples were analyzed using a timsTOF Pro mass spectrometer (Bruker Daltonics) coupled online to an Evosep One liquid chromatography system (Evosep). The peptides were loaded onto a C18-reversed phase analytical column (15 cm long, 150 μm inner diameter, 1.9 μm resin) in buffer A (0.1% formic acid in water) and separated with a linear gradient of buffer B (99.9% acetonitrile and 0.1% formic acid) at a flow rate of 220 nl/min.

The timsTOF Pro was operated in positive ion mode using the parallel accumulation–serial fragmentation acquisition method. The electrospray voltage was set to 1.6 kV. The ion mobility separation range was set from 0.75 to 1.35 V s/cm^2^. Each acquisition cycle consisted of 1 mass spectrometry (MS) scan followed by eight parallel accumulation–serial fragmentation tandem mass spectrometry scans. Active exclusion of previously sequenced precursors was enabled with a release time of 24 s. Precursors and fragments were acquired over a mass range of *m/z* 100 to 1700 using the TOF detector.

### Database Search and Protein Quantification

Raw MS data were processed and searched using MaxQuant software (version 1.6.14; https://www.maxquant.org/). Tandem mass spectra were queried against the UniProt protein database (SwissProt_Homo_sapiens_20395_20210106.fasta), which contains 20,370 protein entries. Database searches were performed using the built-in Andromeda search engine with the following parameters: Trypsin was used as the digestion enzyme, allowing up to two missed cleavages. The first search mass tolerance for precursor ions was set at 20 ppm, followed by a main search tolerance of 6 ppm. The mass tolerance for fragment ions was set at 20 ppm.

Carbamidomethylation of cysteine was set as a fixed modification, while oxidation of methionine was considered a variable modification. The false discovery rate was controlled at 1% at both the peptide and protein levels. For protein quantification, label-free quantification (LFQ) was performed using the MaxLFQ algorithm with razor and unique peptides included. The minimum LFQ ratio count was set to 1, and the “match between runs” function was enabled with a match time window of 2 min to transfer identifications across adjacent runs.

### Experimental Design and Statistical Rationale

A total of 10 biological samples were analyzed in this label-free quantitative proteomic study, including three biological replicates for MTR4 RNAi and two biological replicates for Act.D-treated samples. Each biological replicate represented an independently collected sample, encompassing both cell collection and nucleoli extraction.

For the MTR4 RNAi experiment, three biological replicates per condition were used for identifying differentially expressed proteins. For Act.D-treated experiments, two biological replicates were assayed and then were compared with the data reported in the previous research (1). Data normalization and statistical analyses were performed using MaxQuant and Perseus software platforms (https://maxquant.net/perseus/). Only proteins that have a fold change of less than 0.7 or greater than 1.43 among all replicates were retained for downstream analysis.

## Results

### Knockdown of MTR4 Affects the Nucleolar Proteome

The nuclear RNA helicase MTR4 has been reported to interact with early ribosome biogenesis factors involved in ITS1 processing (45). Additionally, MTR4 plays a crucial role in rRNA processing within the nucleolus, further highlighting its importance in ribosome biogenesis and RNA metabolism. Therefore, MTR4 is critical for the maintenance and regulation of nucleolar functions. The nucleolar proteome, including MTR4, is highly dynamic and undergoes substantial changes in response to cellular stresses. These dynamics are closely linked to the regulation of ribosome biogenesis and RNA metabolism, processes in which MTR4 is deeply involved. Combining nucleolar isolation with advanced quantitative proteomics provides a robust platform for uncovering the molecular events underlying nucleolar dynamics (8, 11, 13). To better understand the nucleolar proteome dictated by MTR4, we conducted quantitative proteomics of HeLa cell nucleoli using mass spectrometry.

**Fig. 1:**
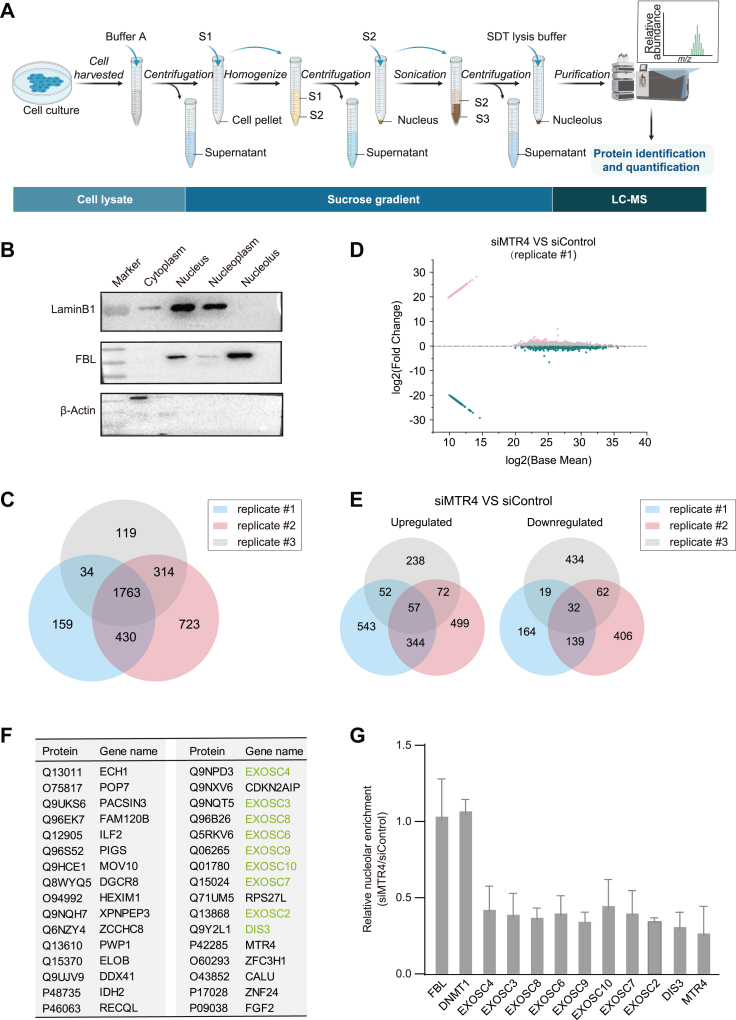
**Identification of nucleolar proteome induced by MTR4 knockdown via combining fractionation with mass spectrometry.***A*, schematic representation of the workflow for nucleoli isolation and subsequent mass spectrometry analysis. *B*, HeLa cells were fractionated into cytoplasmic, nuclear, nucleoplasmic, and nucleolar fractions, followed by Western blotting to examine the purity of the nucleolar fractions. β-Actin was used as a marker for cytoplasmic fractions, fibrillarin for nucleolar fractions, and laminB1 for nucleoplasmic fractions. *C*, the Venn diagram showed 1763 overlapping genes identified across the three replicates involving control cell nucleoli. *D*, nucleolar proteome obtained from HeLa cells following 72 h RNAi targeting MTR4 was analyzed using the M-versus-A plot. *E*, the Venn diagrams showed 57 proteins that are upregulated (*left*) and 32 proteins (*right*) that are downregulated across all three samples. Fold changes higher than 1.43-fold are considered upregulated, and less than 0.7-fold are considered downregulated. *F*, list of downregulated nucleolar proteins after MTR4 RNAi across three independent experiments. *Green* indicates the components of the RNA exosome. *G*, relative protein levels of the RNA exosome subunits in control and MTR4-depleted cells within the nucleolar proteome dataset.

Nucleoli were isolated by sucrose density gradient centrifugation following a previously established procedure (1, 46) (Fig. 1*A*). The purity of the isolated nucleoli was verified by Western blotting (Fig. 1*B*, Supplemental Fig. S1, *A* and *B*). In each nucleolar proteomics experiment, fibrillarin (FBL), NPM1 (B23), UBTF (UBF), and DDX21 were consistently identified by mass spectrometry. Mitochondrial proteins were used as controls, which are barely detected (Supplemental Fig. S1*C*). Across three independent experiments, approximately 1763 nucleolar proteins were consistently detected (Fig. 1*C*). A comparison with previously reported datasets (1) identified 432 overlapping proteins in the nucleolar proteome (Supplemental Fig. S1*D*) (Supplemental Table S1). These data indicated that this method was a reproducible and robust way to quantify the nucleolar proteome.

To explore the role of MTR4 in regulating the nucleolar proteome, we conducted proteomic experiments using purified nucleoli isolated from both control and MTR4 knockdown HeLa cells. Using fold-change thresholds of <0.7 or >1.43, we identified differentially accumulated nucleolar proteins in MTR4 knockdown *versus* control samples. While this stringent criterion improves the robustness and reproducibility of the analysis, it may also exclude some true-positive candidates with moderate changes, potentially leading to false negatives. M-versus-A plot analysis demonstrated consistent trends across three biological replicates (Fig. 1*D*, Supplemental Fig. S1*E*). Gene ontology analysis revealed that MTR4-regulated nucleolar proteins predominantly participate in rRNA processing, RNA splicing, and translation (Supplemental Fig. S2*A*). In total, we identified 57 proteins enriched and 32 proteins depleted in the nucleolar proteome following MTR4 RNAi across three biological replicates (Fig. 1*E*, Supplemental Fig. S2*B*).

Specifically, 9 of the 11 RNA exosome complex subunits exhibited reduced nucleolar accumulation (Fig. 1*F*). Each subunit of the RNA exosome complex showed approximately a 50% reduction in nucleolar protein levels, further underscoring the role of MTR4 in regulating the RNA exosome localization (Fig. 1*G*). Our stringent threshold excluded EXOSC1 and EXOSC5 in proteome analysis. While EXOSC1 and EXOSC5 fell below this threshold in two of the three replicates (0.29 and 0.33 for EXOSC1, 0.45 and 0.26 for EXOSC5), their fold changes in the third replicate were marginally above the cutoff (0.77 and 0.74, respectively) (Supplemental Fig. S2*C*). The immunofluorescence staining experiment further validated that the knockdown of MTR4 depleted EXOSC1 and EXOSC5 from the nucleoli (see below). In contrast, certain proteins, such as PTBP2 and CELF1, were enriched in the nucleolar proteome dataset following MTR4 RNAi (Supplemental Fig. S2*B*). PTBP2 and CELF1 are RNA-binding proteins essential for RNA processing and regulation (47, 48, 49). Consistent with these findings, immunofluorescence staining in HeLa cells also revealed significant enrichment of PTBP2 and CELF1 in the nucleolus following MTR4 RNAi (Supplemental Fig. S3, *A* and *D*), which was further confirmed by Western blotting (Supplemental Fig. S3, *B* and *E*). PTBP2 and CELF1 protein levels remained largely unchanged in whole-cell lysates (Supplemental Fig. S3, *C* and *F*).

Together, these data revealed that MTR4 plays a critical role in modulating the nucleolar proteome, particularly regarding the RNA exosome complex in the nucleolus.

### EXOSC10 is Enriched in the GC Region of the Nucleolus

**Fig. 2:**
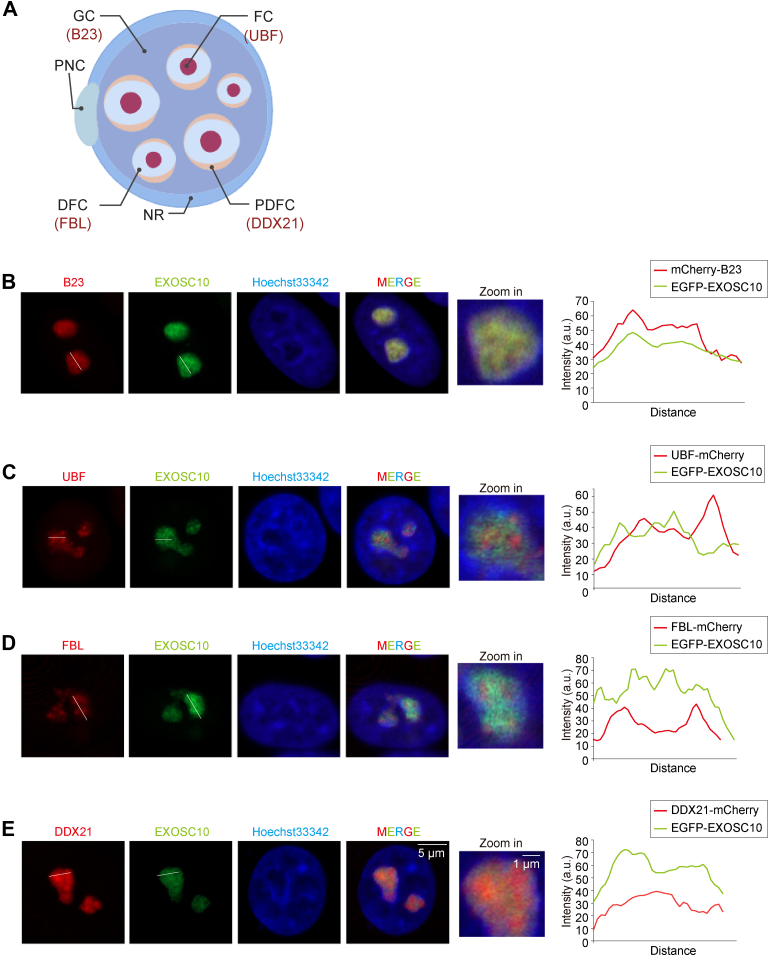
**EXOSC10 is enriched in the GC region in the nucleolus.***A*, the schematic depicts the different subregions of the nucleolus. A mammalian nucleolus comprises four subregions: FC, DFC, PDFC, and GC. FC refers to the fibrillar center, DFC to the dense fibrillar component, PDFC to the periphery of the dense fibrillar component, GC to the granular component, and NR to the nucleolar rim. *B*–*E*, representative images show the localization of B23, UBF, FBL, or DDX21 (*red*) and EXOSC10 (*green*). EXOSC10 colocalized with B23. A fluorescent density scan of nucleolar proteins and EXOSC10 staining was performed using ImageJ (https://imagej.net/ij/). The *white lines* indicate the areas where fluorescence quantification was conducted. FBL, fibrillarin.

To further validate the regulation of MTR4 on the subcellular localization of the RNA exosome complex, we established stable cell lines expressing an enhanced green fluorescent protein (EGFP)-EXOSC10 fusion in HeLa and 293T cells, referred to as HeLa(EGFP-EXOSC10) and 293T(EGFP-EXOSC10), respectively. To label nucleolar subregions, we constructed plasmids expressing mCherry-tagged nucleolar markers: UBF for FC, FBL for DFC, DDX21 for PDFC, and B23 for GC (Fig. 2*A*). Subsequently, HeLa(EGFP-EXOSC10) cells were transfected with these mCherry-tagged nucleolar markers to examine the colocalization of EGFP-EXOSC10 with these markers using confocal microscopy. EXOSC10 was highly enriched in the nucleolus, displaying strong colocalization with B23 and partial colocalization with FBL, UBF, and DDX21 (Fig. 2, *B*–*E*). B23-marked GC region is implicated in late-stage pre-rRNA processing and rRNA ribonucleoprotein assembly, consistent with the role of EXOSC10 in pre-rRNA processing.

### The Knockdown of MTR4 Depletes EXOSC10 from the Nucleolus

**Fig. 3:**
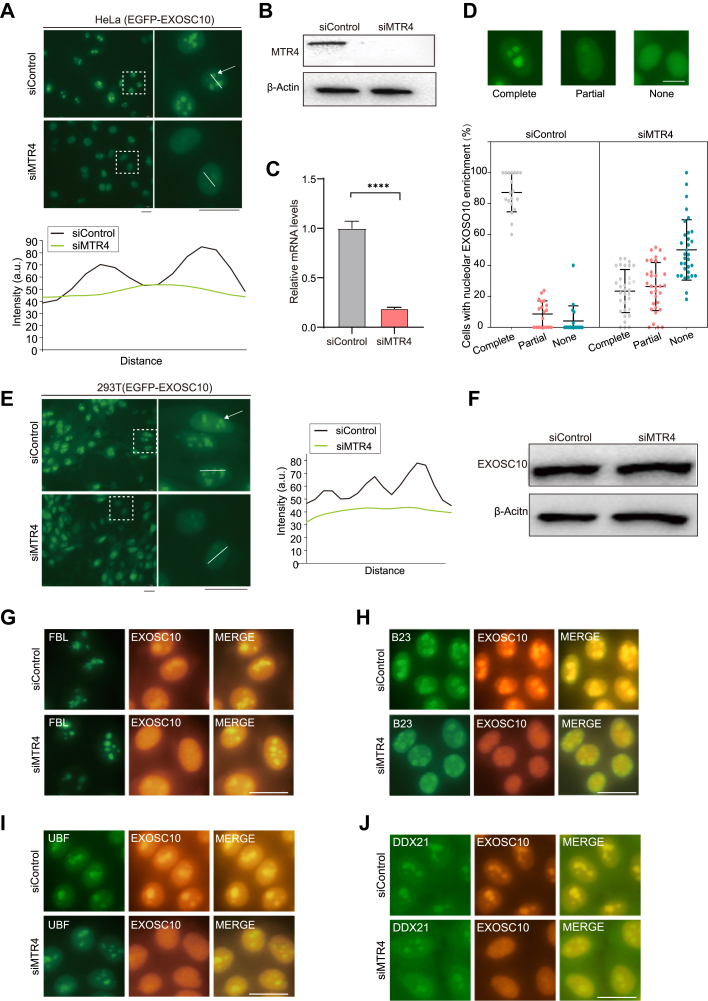
**Depletion of MTR4 triggers RNA exosome translocation from the nucleolus to the nucleoplasm.***A*, images show that the depletion of MTR4 by RNAi results in EGFP-EXOSC10 translocation from the nucleoli to the nucleoplasm in HeLa cells. The *white lines* indicate the area where fluorescence quantification was performed. The scale bar represents 20 μm. *B*, the efficiency of MTR4 depletion was analyzed using Western blotting with the indicated antibodies. *C*, RT-qPCR was conducted to validate the mRNA knockdown efficiency in MTR4-KD cells. Mean ± SD; n = 3. *D*, the percentage of relative EXOSC10 distribution in the nucleolus and nucleoplasm following 72 h RNAi targeting MTR4. The data were calculated from independent microscopic images (n = 3; total cell number: 500–700). The scale bar represents 10 μm. *E*, images show that MTR4 depletion results in EGFP-EXOSC10 translocation from the nucleolus to the nucleoplasm in 293T(EGFP-EXOSC10) cells. The *white arrows* indicate EXOSC10. The *white lines* indicate the area where fluorescence quantification was performed. The scale bar represents 20 μm. *F*, Western blot analysis revealed that the expression of endogenous EXOSC10 was not noticeably affected following MTR4 knockdown via siRNA transfection for 72 h. A non-targeting siRNA was used as a control. *G*–*J*, immunofluorescence staining of MTR4-KD HeLa cells with antibodies against FBL (*G*), B23 (*H*), UBF (*I*), DDX21 (*J*), and EXOSC10 was performed 72 h later after transfection with MTR4 siRNA. The scale bar represents 20 μm. FBL, fibrillarin; MTR4 KD, MTR4 knockdown.

To confirm the proteomic results, we transfected MTR4-targeting siRNA into HeLa(EGFP-EXOSC10) cells. As expected, the knockdown of MTR4 specifically led to the depletion of EGFP-EXOSC10 from the nucleolus (Fig. 3, *A*–*C*). We quantified the relative enrichment of EGFP-EXOSC10 in the nucleolus in control and MTR4 RNAi-treated cells, revealing a marked reduction in the nucleolar enrichment of EXOSC10 following MTR4 knockdown (Fig. 3*D*). Similarly, the knockdown of MTR4 by RNAi in 293T(EGFP-EXOSC10) cells resulted in the translocation of EGFP-EXOSC10 from the nucleolus to the nucleoplasm (Fig. 3*E*). We performed immunofluorescence staining with the anti-EXOSC10 antibody in MTR4-depleted HeLa cells and found that endogenous EXOSC10 proteins were also depleted from the nucleolus (Supplemental Fig. S4*A*). The expression levels of both EXOSC10 and EGFP-EXOSC10 were not significantly affected by MTR4 RNAi treatment (Fig. 3*F*, Supplemental Fig. S4*B*). Notably, MTR4 depletion did not noticeably alter nucleolar morphology and integrity, as shown by immunostaining with anti-FBL, anti-B23, anti-UBF, and anti-DDX21 antibodies (Fig. 3, *G*–*J*). Knockdown of EXOSC10 by RNAi in HeLa(EGFP-MTR4) cells did not affect MTR4 localization (Supplemental Fig. S4, *C* and *D*).

To assess whether the alteration in EXOSC10 localization affects the mobility of nucleolar proteins, we performed a fluorescence recovery after photobleaching assay. The depletion of MTR4 by RNAi did not significantly change the mobility of mCherry-B23 and FBL-mCherry (Supplemental Fig. S4, *E* and *F*). Collectively, the knockdown of MTR4 induces the relocalization of EXOSC10 to the nucleoplasm without compromising nucleolar integrity.

### MTR4 Specifically Regulates the Nucleolar Accumulation of the RNA Exosome

**Fig. 4:**
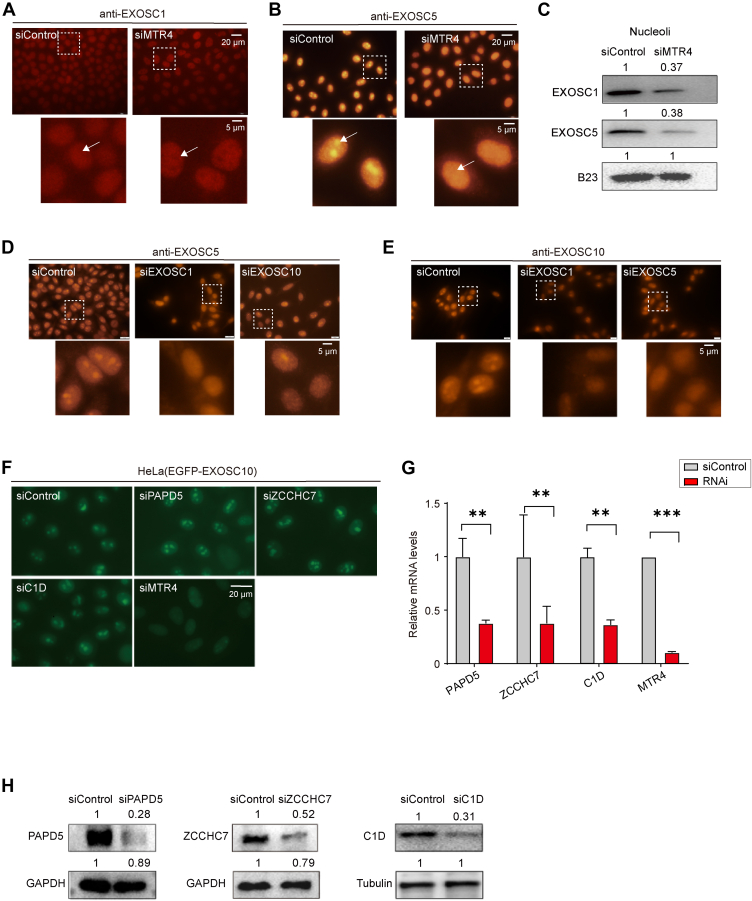
**The RNA exosome mislocalization is specifically regulated by MTR4.***A* and *B*, HeLa cells were immunostained with EXOSC1 and EXOSC5 antibodies following 72 h of RNAi targeting MTR4. The *white arrows* indicate the nucleolus. *C*, Western blot analysis of EXOSC1 and EXOSC5 in nucleolar fractions after MTR4 knockdown (KD). The expression levels of these proteins in the nucleolus are quantified by ImageJ. *D*, immunostaining of EXOSC5 in HeLa cells after transfection with EXOSC1 or EXOSC10 siRNAs for 72 h. *E*, immunostaining of EXOSC10 in HeLa cells after transfection with EXOSC1 or EXOSC5 siRNAs for 72 h. *F*, images of HeLa(EGFP-EXOSC10) cells showed that the depletion of TRAMP component by RNAi did not affect the enrichment of EXOSC10 in the nucleolus. *G*, RT-qPCR analysis was performed to assess the knockdown efficiency of TRAMP complex components in siRNA-treated cells. Mean ± SD; n = 3. *H*, the efficiency of TRAMP complex components depletion was analyzed using Western blotting with the indicated antibodies. EGFP, enhanced green fluorescent protein; TRAMP, Trf4/5-Air1/2-Mtr4 polyadenylation.

Similar to EXOSC10, immunofluorescence staining also revealed that both EXOSC1 and EXOSC5 were dispersed from the nucleolus to the nucleoplasm in MTR4-depleted HeLa cells (Fig. 4, *A* and *B*, Supplemental Fig. S5, *A* and *B*). Western blot analysis indicated that MTR4 depletion resulted in approximately 60% reduction in the nucleolar levels of EXOSC1 and EXOSC5 (Fig. 4*C*). Previously isolated mutations in the RNA exosome core subunit Rrp43p have been shown to negatively affect the function of the complex in yeast (50, 51). To further investigate whether the nucleolar localization of RNA exosome subunits is mutually dependent on other exosome components in HeLa cells, we examined the effects of RNAi-mediated knockdown of the exosome subunits. EXOSC5, which is primarily enriched in the nucleolus, was depleted from the nucleolus upon knockdown of EXOSC1 or EXOSC10 (Fig. 4*D*, Supplemental Fig. S5*C*). Similarly, RNAi targeting EXOSC1 or EXOSC5 resulted in the depletion of EXOSC10 from the nucleolus (Fig. 4*E*, Supplemental Fig. S5*D*). Notably, neither MTR4 depletion nor the depletion of RNA exosome subunits led to a gross decrease in EXOSC1 or EXOSC5 levels in the whole-cell lysate (Supplemental Fig. S5*E*).

MTR4 is involved in the composition and function of several complexes, including TRAMP, PAXT, and NEXT complexes. We examined whether the localization of the RNA exosome is dependent on these three complexes. RNAi knockdown of the TRAMP, PAXT, and NEXT complexes did not significantly affect the levels of nucleolar EXOSC10. Additionally, knockdown of C1D, an RNA exosome-associated factor, did not disturb the nucleolar localization of EXOSC10 either (Fig. 4, *F*–*H*, Supplemental Fig. S6, *A–C*). Additionally, to investigate whether the translocation of the RNA exosome was caused by mRNA accumulation in the nucleus due to MTR4 depletion, we utilized RNAi to knock down several components of the TREX complex, including ALYREF, UAP56, and THOC2, in HeLa(EGFP-EXOSC10) cells. However, no apparent effect on the levels of nucleolar EXOSC10 was observed (Supplemental Fig. S6, *D*–*F*). In summary, these data suggest that the enrichment of the nucleolar RNA exosome is specifically modulated by MTR4.

### Identification of Chemicals that Modulate the RNA Exosome Nucleolar Accumulation

To identify additional factors governing the RNA exosome localization, we performed a candidate-based RNAi screen in HeLa(EGFP-EXOSC10) cells, targeting approximately 60 genes involved in RNA processing and ribosome biogenesis (Supplemental Table S2). Interestingly, only MTR4 knockdown specifically caused the depletion of EGFP-EXOSC10 from the nucleolus.

**Fig. 5:**
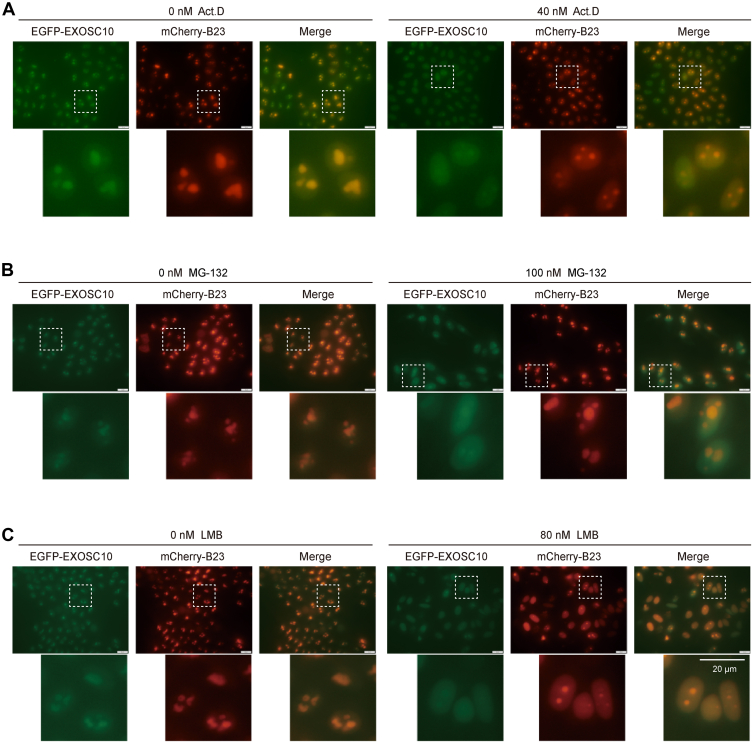
**Identification of chemicals modulating EXOSC10 nucleolar accumulation.***A*, Act. D, (*B*) MG-132, and (*C*) LMB treatments induced the depletion of EGFP-EXOSC10 from the nucleolus. The scale bar represents 20 μm. Act.D, actinomycin D; EGFP, enhanced *green* fluorescent protein; LMB, leptomycin B.

To explore whether other cellular processes also affect the localization of the RNA exosome, we treated HeLa(EGFP-EXOSC10) cells with several chemicals and examined the nucleolar structure as well as the distribution of the RNA exosome. We observed that treatment with Act.D, MG-132, and LMB induced the depletion of EXOSC10 from the nucleolus. Act.D inhibits RNA polymerase I transcription elongation and induces nucleolar segregation (40, 52). We found that Act.D treatment led to the disintegration of the nucleolus and the depletion of EXOSC10 within the nucleolus (Fig. 5*A*). MG-132 blocks the proteolytic activity of the 26S proteasome complex and triggers apoptosis (53). Treatment with MG-132 resulted in the dispersal of EXOSC10 throughout the nucleus but did not affect the shape or integrity of the nucleolus (Fig. 5*B*). LMB, an inhibitor of nuclear export, induces the nuclear accumulation of proteins that shuttle between the cytoplasm and nucleus (54). LMB treatment resulted in nucleolar disintegration and also the dispersion of EXOSC10 within the nucleus (Fig. 5*C*).

However, FCCP (an oxidative phosphorylation uncoupler), or CHX (a protein synthesis inhibitor) did not affect the nucleolar localization of EXOSC10 (Supplemental Fig. S7, *A* and *B*) (55). To investigate whether culturing condition influences the localization of the RNA exosome in HeLa cells, we removed FBS from the culture medium and found that serum starvation did not affect the nucleolar enrichment of EXOSC10 (Supplemental Fig. S7*C*).

### Proteomic Analysis of Act.D-Treated Nucleoli Revealed that RNA Exosome Distribution Depends on the Nucleolar Localization Sequence (NoLS) of MTR4

**Fig. 6:**
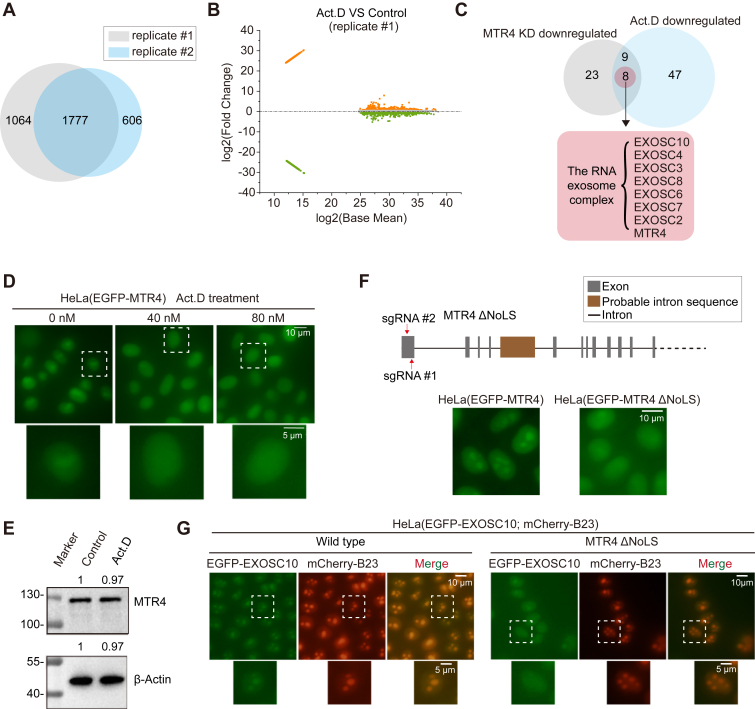
**Act.D treatment depletes the RNA exosome components from the nucleolus through modulating nucleolar MTR4.***A*, the Venn diagram showed 1777 overlapping genes identified across the two replicates involving nucleoli isolated from HeLa cells treated with 40 nM Act.D for 1 h. *B*, M-versus-A plot identified differential proteins in nucleolar proteome following Act.D treatment. *C*, the overlapping analysis identified downregulated nucleolar proteins both in MTR4 knockdown (KD) and Act.D-treated cells. *D*, EGFP-MTR4 dispersed to the nucleoplasm in HeLa(EGFP-MTR4) cells treated with 40 nM or 80 nM Act.D. *E*, Western blot analysis showed the protein levels of MTR4 in whole-cell lysate after Act.D treatment in HeLa cells. The levels of these proteins in the nucleolus were quantified by ImageJ. *F*, *Top*: schematic representation of the gene structure of MTR4. Sequence predictions suggest that the nucleolar localization sequence of MTR4 is located in the first exon. *Bottom*: deletion of the NoLS from MTR4 using CRISPR/Cas9 technology in HeLa(EGFP-MTR4) cells led to impaired nucleolar localization of EGFP-MTR4. The *red lines* indicate the targeted regions of sgRNA1 and sgRNA2. *G*, the nucleolar localization of MTR4 was required for the nucleolar accumulation of EXOSC10. A double fluorescence tagged cell line, HeLa(EGFP-EXOSC10; mCherry-B23), was constructed, and the nucleolar localization sequence (NoLS) of MTR4 was then disrupted by CRISPR/Cas9 technology. Act.D, actinomycin D.

To compare the nucleolar proteome alterations induced by Act.D treatment with MTR4 knockdown, we isolated the Act.D-treated nucleoli from HeLa cells and performed proteomic analysis. In Act.D-treated nucleoli, 1777 proteins were identified across two replicates (Fig. 6*A*). M-versus-A plot analysis identified differentially expressed proteins between the Act.D-treated and control samples (Fig. 6*B*, Supplemental Fig. S8*A*) (Supplemental Table S1). A total of 506 differential proteins were identified in two biological replicates, including 151 downregulated and 220 upregulated proteins, using thresholds of <0.7-fold for downregulation and >1.43-fold for upregulation, respectively (Supplemental Fig. S8*B*). Factors that were depleted from nucleolus after Act.D treatment included ribosomal proteins, RNA processing factors, and small subunit processome components (Supplemental Fig. S8*C*). Although the nucleolar proteomes differed between MTR4-knockdown and Act.D-treated nucleoli, the RNA exosome displayed similar reduction in both treatments (Fig. 6*C*, Supplemental Fig. S8*D*).

We observed that Act.D treatment reduced nucleolar MTR4 levels (Fig. 6*D*, Supplemental Fig. S8*E*) without affecting gross cellular MTR4 levels (Fig. 6*E*), suggesting a potential link between MTR4 nucleolar levels and the RNA exosome distribution. To test whether the Act.D-induced localization change of the RNA exosome is at least partially due to its effect on the nucleolar accumulation of MTR4, we disrupted the nucleolar localization sequence (NoLS) of MTR4 using CRISPR/Cas9 technology. As expected, the loss of the NoLS disrupted the nucleolar accumulation of MTR4 (Fig. 6*F*). Consistently, the disruption of the MTR4 NoLS also led to a reduced nucleolar EGFP-EXOSC10 level (Fig. 6*G*), revealing a critical role of the nucleolar localization sequence (NoLS) of MTR4 in regulating the nucleolar accumulation of the RNA exosome. Taken together, these results suggest that Act.D may influence the nucleolar accumulation of the RNA exosome by regulating nucleolar MTR4 levels.

## Discussion

In this study, we analyzed nucleolar proteomes by isolating nucleoli from MTR4-knockdown HeLa cells followed by mass spectrometry. We found that MTR4 knockdown reshaped the nucleolar proteomes and regulated the nucleolar localization of the RNA exosome complex, which also depends on the integrity of its components. Additionally, we identified several chemicals, including Act.D, which regulate the subcellular localization of the RNA exosome. Both MTR4 knockdown and Act.D treatment differentially reshaped the nucleolar proteomes but similarly reduced the nucleolar accumulation of the RNA exosome complex. Act.D treatment also reduced the level of MTR4 within the nucleolus. Knocking out the NoLS of MTR4 impaired the RNA exosome localization. These findings suggest that the nucleolar localization of MTR4 contributes to the subcellular localization of the RNA exosome. Overall, our work demonstrates that both MTR4 and rRNA transcription are crucial for maintaining nucleolar proteomes and the nucleolar localization of the RNA exosome complex.

The RNA exosome interacts with compartment-specific cofactors and substrates, consistent with their nuclear RNA processing roles (21, 22). In yeast, all RNA exosome subunits predominantly localize to the nucleolus. Notably, in agreement with findings in yeast (22), we found that the catalytic subunit EXOSC10 is enriched in the GC region in HeLa cells, where intermediate rRNA processing occurs. Moreover, our results raise intriguing questions regarding the molecular mechanisms that determine the precise subnucleolar localization of EXOSC10. In *Saccharomyces cerevisiae*, the karyopherins Srp1, Kap95, and Sxm1 have been shown to mediate the nuclear import of Rrp6 by recognizing distinct nuclear localization signals (56). These findings suggest that specific nuclear transport receptors, together with defined sequence elements in EXOSC10, may coordinate its nuclear import and subnucleolar targeting in cells. Future investigations into these pathways will provide valuable insights into the regulatory network governing the RNA exosome compartmentalization and function in higher eukaryotes.

Dynamic changes in subcellular localization enable cells to respond efficiently to intracellular and environmental stimuli. For example, nucleolin is a multifunctional protein primarily localized in the nucleolus but is also found in the nucleoplasm, cytoplasm, and cell membrane (57, 58). The mobilization of nucleolin from the nucleolus to the perinuclear region may enhance viral mRNA translation (59). Moreover, among the large family of HSPs, HSP70s translocate into the nucleus during cellular stress and return to the cytoplasm after several hours of recovery (60). This process is essential for disassembling stress granules in both the cytoplasm and the nucleus. It also facilitates anisosomes formation and helps transport aggregation-prone proteins to the nucleolus for proteasomal degradation during heat shock (6, 61, 62, 63). Similarly, EXOSC10 can relocate from the nucleolus to DNA damage sites to recruit RAD51 and facilitate homologous recombination (41), suggesting that specific protein translocation is related to protecting cells from environmental stress and contributes to cellular homeostasis.

The RNA exosome plays a key role in regulating essential cellular functions by catabolizing various RNA species in different subcellular compartments (64, 65). Upon exposure to multiple stressors, EXOSC10 can relocate to specific subcellular compartments. For instance, treatment with Act.D leads to the dispersal of EXOSC10 and the disassembly of the nucleolus. After the removal of Act.D from the culture medium, EXOSC10 relocalized to the nucleolus. Act.D inhibits Pol I transcription elongation, resulting in the inactivation of rRNA processing and maturation, which ultimately leads to nucleolar segregation (52, 66, 67). Additionally, Act.D disrupts nucleolar structure, causing the nucleolar proteins, including FBL, NPM1, and the RNA exosome subunit EXOSC10, to passively diffuse into the cytoplasm. After the removal of Act.D, the nucleolar structure is restored, and EXOSC10, along with other proteins, is transported back to the nucleolus.

The localization of the RNA exosome depends on the presence of MTR4 in the nucleolus. However, the knockdown of EXOSC10 does not affect the localization of MTR4. The unidirectional regulation of EXOSC10 by MTR4 suggests that MTR4 functions upstream of the RNA exosome complex. MTR4 is a shared subunit of the TRAMP, NEXT, and PAXT complexes, but no other subunits of these complexes appear to affect RNA exosome enrichment in the nucleolus, implying a specific role for MTR4.

Errors in ribosome biogenesis are detected in the nucleolus, leading to global changes in nucleolar function and morphology (68). The nucleolar RNA exosome is essential for ribosome biogenesis, including rRNA synthesis, processing, and ribosome assembly (69). The primary roles of MTR4 and the RNA exosome involve the 3′ processing of rRNA and the regulation of RNA stability (70). We hypothesize that the depletion of MTR4 may impair rRNA maturation, hinder the degradation of erroneous rRNAs, and disrupt ribosome assembly. Defects in rRNA degradation result in the excessive accumulation of erroneous rRNAs in the nucleolus. This overload prevents the nucleolus from efficiently managing the erroneous rRNAs, leading to their translocation to the nucleoplasm. In response, the RNA exosome complex relocates from the nucleolus to the nucleoplasm, accompanied by these defective rRNAs. Further investigations are needed to determine whether forced mislocalization of the RNA exosome components could provoke distinct biological responses.

Recently, mutations in genes encoding both structural and catalytic subunits of the RNA exosome have been linked to human diseases (71). The RNA exosome complex can degrade expanded hexanucleotide repeat RNA in C9orf72 frontotemporal lobar degeneration (FTLD)/amyotrophic lateral sclerosis (ALS) patients. Frequent mislocalization of EXOSC10 has been observed in cells expressing arginine-rich dipeptide repeat protein in a RAN translation-dependent manner, suggesting that the mislocalization of EXOSC10 may disrupt its functionality (72). Additionally, nucleolar dipeptide repeat protein may disrupt nucleolar quality control, contributing to cellular pathology in ALS and FTLD patients (6). It is worth exploring whether the localization of the RNA exosome can serve as a marker for early detection of ALS and FTLD in patients in the future.

Dynamic alterations in subcellular localization enable cells to swiftly respond to diverse stimuli. Therefore, the subcellular fractionation technique, combined with confocal microscopy and molecular studies, provides a powerful tool for understanding how the nucleolus regulates protein function by controlling protein entry and exit under different growth conditions. It also marks a significant advancement in studying the coordinated roles of various complexes. Meanwhile, we employed a proteomic approach and identified that the depletion of MTR4 induced the nucleolar accumulation of certain RNA processing–related proteins, such as PTBP2 and CELF1. Furthermore, combining mass spectrometry with nucleolar fractionation will provide important new insights into how and why the nucleolar proteome is altered in response to different environmental stimuli and developmental conditions.

### Statistics

The means and SDs of the results are presented in bar graphs with error bars. Statistical analysis was performed using two-tailed Student’s *t*-tests.

## Data Availability

The data that support this study are available from the corresponding author upon request. The mass spectrometry proteomics data have been deposited to the ProteomeXchange Consortium (https://proteomecentral.proteomexchange.org) via the iProX partner repository with the dataset identifier PXD060006.

All annotated mass spectrometry data from this study have been deposited in MS-Viewer for public access. The annotated spectra can be accessed via the following URLs:

Rep#1 Control and MTR4 KD:

Search key: bntkldrt38

URL: https://msviewer.ucsf.edu/prospector/cgi-bin/mssearch.cgi?report_title=MS-Viewer&search_key=bntkldrt38&search_name=msviewer

Rep#2 Control and MTR4 KD:

Search key: t4utnkpmmo

URL: https://msviewer.ucsf.edu/prospector/cgi-bin/mssearch.cgi?report_title=MS-Viewer&search_key=t4utnkpmmo&search_name=msviewer

Rep#3 Control and MTR4 KD:

Search key: mv9tfhtueb

URL: https://msviewer.ucsf.edu/prospector/cgi-bin/mssearch.cgi?report_title=MS-Viewer&search_key=mv9tfhtueb&search_name=msviewer

Rep#1 Control and Act.D:

Search key: wwnqemsn2o

URL: https://msviewer.ucsf.edu/prospector/cgi-bin/mssearch.cgi?report_title=MS-Viewer&search_key=wwnqemsn2o&search_name=msviewer

Rep#2 Control and Act.D:

Search key: qcxbwzu0es

URL: https://msviewer.ucsf.edu/prospector/cgi-bin/mssearch.cgi?report_title=MS-Viewer&search_key=qcxbwzu0es&search_name=msviewer.

## Supporting Information

This article contains supporting information (1).

## Conflict of Interest

The authors declare no competing interests.
