# Supplementary material for: Nucleolar Proteomics Revealed the Regulation of RNA Exosome Localization by MTR4
Source: Mol Cell Proteomics. 2025 Jul 10;24(8):101031. doi: 10.1016/j.mcpro.2025.101031 (PMC12356310; doi:10.1016/j.mcpro.2025.101031)
Supplement: Table S2 [file mmc2.docx]

**Table S2: List of genes tested in the candidate-based RNAi screen.**

| **Proteins** | **RNAi** | **EXOSC10 localization** |
| --- | --- | --- |
| MTR4 | siRNA | Nucleoli, Nucleoplasm |
| EXOSC1 | siRNA | Nucleoli, Nucleoplasm |
| EXOSC5 | siRNA | Nucleoli, Nucleoplasm |
| ALYREF | siRNA | Nucleoli |
| C1D | siRNA | Nucleoli |
| CELF1 | siRNA | Nucleoli |
| PAPD5 | siRNA | Nucleoli |
| PTBP2 | siRNA | Nucleoli |
| RBM7 | siRNA | Nucleoli |
| RPL27 | siRNA | Nucleoli |
| RPL28 | siRNA | Nucleoli |
| RPS11 | siRNA | NA |
| RPS2 | siRNA | NA |
| RPS25 | siRNA | Nucleoli |
| RPS27 | siRNA | Nucleoli |
| RPS5 | siRNA | NA |
| RPS6 | siRNA | NA |
| RRP36 | siRNA | Nucleoli |
| THOC2 | siRNA | Nucleoli |
| UAP56 | siRNA | Nucleoli |
| ZCCHC7 | siRNA | Nucleoli |
| ZCCHC8 | siRNA | Nucleoli |
| ZFC3H1 | siRNA | Nucleoli |
| ZNF277 | siRNA | Nucleoli |
| CBX6 | shRNA | Nucleoli |
| CBX8 | shRNA | Nucleoli |
| CHD1 | shRNA | Nucleoli |
| CHD2 | shRNA | Nucleoli |
| CHD4 | shRNA | Nucleoli |
| DDX31 | shRNA | Nucleoli |
| DDX3X | shRNA | Nucleoli |

**Continued:**

| **Proteins** | **RNAi** | **EXOSC10 localization** |
| --- | --- | --- |
| DDX5 | shRNA | Nucleoli |
| DDX54 | shRNA | Nucleoli |
| FDFT1 | shRNA | Nucleoli |
| FKBP8 | shRNA | Nucleoli |
| GANAB | shRNA | Nucleoli |
| GTPBP4 | shRNA | Nucleoli |
| HSPA1A | shRNA | Nucleoli |
| HSPA1B | shRNA | Nucleoli |
| HYOU1 | shRNA | Nucleoli |
| KAT5 | shRNA | Nucleoli |
| PABPN1 | shRNA | Nucleoli |
| PDCD6IP | shRNA | Nucleoli |
| POP1 | shRNA | Nucleoli |
| RPP14 | shRNA | Nucleoli |
| RPP25 | shRNA | Nucleoli |
| RPP30 | shRNA | Nucleoli |
| RRN3 | shRNA | Nucleoli |
| SCRIB | shRNA | Nucleoli |
| SLC25A12 | shRNA | Nucleoli |
| SNRPA | shRNA | Nucleoli |
| TAF12 | shRNA | Nucleoli |
| UBR5 | shRNA | Nucleoli |
| UBTF | shRNA | Nucleoli |
| UGGT1 | shRNA | Nucleoli |
| XRCC5 | shRNA | Nucleoli |
