# Supplementary material for: Nucleolar Proteomics Revealed the Regulation of RNA Exosome Localization by MTR4
Source: Mol Cell Proteomics. 2025 Jul 10;24(8):101031. doi: 10.1016/j.mcpro.2025.101031 (PMC12356310; doi:10.1016/j.mcpro.2025.101031)
Supplement: Table S3 [file mmc3.docx]

**Table S3: Expression plasmids used in this study, related to experimental procedures.**

| **Plasmids Name** | **Relevant information** |
| --- | --- |
| pPBmL-PNEE-EGFP-EXOSC10 | AmpR, EF1α promoter, Neomycin |
| pPBmL-PNEE-EGFP-MTR4 | AmpR, EF1α promoter, Neomycin |
| pPBmL-PNEE-DDX21-mCherry | AmpR, EF1α promoter, Neomycin |
| pcDNA3.1-mCherry-B23 | KanR, CMV promoter, Neomycin |
| pcDNA3.1-FBL-mCherry | KanR, CMV promoter, Neomycin |
| pcDNA3.1-UBF-mCherry | KanR, CMV promoter, Neomycin |
