# Supplementary material for: Nucleolar Proteomics Revealed the Regulation of RNA Exosome Localization by MTR4
Source: Mol Cell Proteomics. 2025 Jul 10;24(8):101031. doi: 10.1016/j.mcpro.2025.101031 (PMC12356310; doi:10.1016/j.mcpro.2025.101031)
Supplement: Table S4 [file mmc4.docx]

**Table S4: siRNA target sequences used for RNAi experiments.**

| **Gene name** | **Sequence** |
| --- | --- |
| Control | UGGUUUACAUGUCGACUAA  UGGUUUACAUGUUGUGUGA  UGGUUUACAUGUUUUCUGA  UGGUUUACAUGUUUUCCUA |
| ALYREF | UCUCAGACGCCGAUAUUCA  GUUAAACAGACCAGCAAAU  GGAACUCUUUGCUGAAUUU  CAAAACAACUUCCCGACAA |
| C1D | CGAGUAUUUGUCAGCGUUU  UGAUAUUAAGAAAGCGUGA  GGUUGUAUGUUGAUGGAUG  UAUGCUAUCUGUAGGCUGA |
| CELF1 | GAGCCAACCUGUUCAUCUA  ACUCGGGUAUCCAGCAAUA  GCUGUUUAUUGGUAUGAUU  UGAAGAAUGCCGGAUAUUG |
| EXOSC1 | CACUAAAGAAUUCCGGAAA  CCGCAAGGAAGAUGUCCGA  GAUGAAGAGCAGCGAGAAU  CCGAAUUCUUGCAGACCUA |
| EXOSC10 | ACGAAAAGCUCUUGAAUUG  UGAAAGUUACGGAUAUGUA  GAAGUGACAUGUACAUUCU  CCCAUUACCUGCUAUAUAU |
| EXOSC5 | CAACAAGGCCACACUCGAA  UGGACAGCGUGGAACGGAA  CGCCAAAAUCCGUGCUGAA  CCGUGGUGCUGCAGGUUGU |
| MTR4 | GCACAUACCUCAGCGGGAA  AGGGAAAAGCAGCGUGUAA  UUACAGAGGUUCCGAAGUU  GAAACUGUGUAUACGCUUU |
| PABPN1 | GGAACGGCCUGGAGUCUGA  AGUCAACCGUGUUACCAUA  GAGUCCACCUCCAGGCAAU  UAUCAAAGCUCGAGUCAGG |
| PAPD5 | CAUCAAUGCUUUAUAUCGA  GGACGACACUUCAAUUAUU  GAUAAAGGAUGGUGGUUCA  GAAUAGACCUGAGUUUUCA |

**Continued:**

| **Gene name** | **Sequence** |
| --- | --- |
| PTBP2 | GAGAGGAUCUGACGAACUA  UGACAUGACUUACGUGCAU  GGAACUAGCAACCGAGGAA  AGAAGAGGAUCUACGAACA |
| RBM7 | GGGUGAAGUUGUAUGAUAA  GGGCAACCUUGAAACGAAA  GACCAUCAUUACAGAGGAA  CACCAUCAUCACAGCGUAA |
| RPL27 | CGCCAAGAGAUCAAAGAUA  ACGCAAAGCUGUCAUCGUG  GUAUAACUACAAUCACCUA  GGUUUUAGAUGCUUUGUUU |
| RPL28 | AAUGGAUGGUCGUGCGGAA  AGAGGAAUAAGCAGACCUA  GGACUGAUUCACCGCAAGA  AAGGUGUCGUGGUGGUCAC |
| RPS11 | GAGACUAUCUGCACUACAU  GGGACGUCCAGAUCGGUGA  ACCAAAAGCAGCCGACCAU  AGAACAUCGGUCUGGGCUU |
| RPS2 | UUAAGGAAUCAGAGAUCAU  CGUCAAGACCCACACCAGA  CAUGAUGGCUGGUAUCGAU  AGUGGAUGCCCGUCACCAA |
| RPS25 | GAAAGUCGGCCAAGAAAGA  UGACAAAGCUACCUAUGAU  CAAAGACCCAGUGAACAAA  AAAGAAGAAGUGGUCCAAA |
| RPS27 | GAUGUGAAAUGCCCAGGAU  GGUCUUUAGCCAUGCACAA  GGAAGCAGCACUAAAAGCA  GAAGAAACGCCUGGUGCAG |
| RPS5 | GAACUCCUAUGCCAUUAAG  ACAUUUCCCUGCAGGAUUA  GCUCAUGACUGUGCGCAUC  AAAGCUCAGUGUCCCAUUG |
| RPS6 | GGACGAUGAACGCAAACUU  UAAAGAAGAUGAUGUCCGC  UGGACUGACUGAUACUACA  GAUGCAAAUCUGAGCGUUC |

**Continued:**

| **Gene name** | **Sequence** |
| --- | --- |
| THOC2 | GGCGAAUAUACUCACAUCA  CGCAUAGGCAAGUCAUUUA  GUAUGACCUUGCAGUUCCA  TCAAACAATAGACAGAGCCA |
| UAP56 | GUCUAUCAAGAAGGAUGAA  GUCAUGAUGUUCAGUGCUA  CGUCAGAAGUCCAGCAUGA  AAGCTCTTTGACCTTCTGGA |
| ZCCHC7 | CCAGAUAGCUAAUAACCGA  UCACAUUACCACACGUCAA  GGAUAAACAGUGUGACCGA  GAAGAUGGACCCAGCGGUA |
| ZCCHC8 | GAGCAGGCCGAGAGCGUAA  GGAAUAUUGGUGAACGAUA  UCGGAAUGCUGCUCGAAUA  CGACAUGGAGCUCGAUUCA |
| ZFC3H1 | CGAUAAUAACAGCCAGAUA  GGCUAUGAGAUGUGAUAUA  AAGAAGAGGAUCAGCGGAA  GAACUAGAAUGCAUCAAUA |
| ZNF277 | AACAGCAGCAAGAACGAAA  GCACAUGAUUAUUGAGCAU  ACAUUGGAUUGCCAGACAA  UCAAGUUGGUUGCUGAUUU |
