# Supplementary material for: Nucleolar Proteomics Revealed the Regulation of RNA Exosome Localization by MTR4
Source: Mol Cell Proteomics. 2025 Jul 10;24(8):101031. doi: 10.1016/j.mcpro.2025.101031 (PMC12356310; doi:10.1016/j.mcpro.2025.101031)
Supplement: Table S5 [file mmc5.docx]

**Table S5: sgRNA sequences and primers used for RT-qPCR.**

| **Primer name** | **Sequence (5' to 3') references** |
| --- | --- |
| MTR4 ΔNoLS sg1F | CACCGAGCTGTTCAGCGTGTTCGA |
| MTR4 ΔNoLS sg1R | AAACTCGAACACGCTGAACAGCTC |
| MTR4 ΔNoLS sg2F | CACCGCCTTGTCTGCAGACCCTGG |
| MTR4 ΔNoLS sg2R | AAACCCAGGGTCTGCAGACAAGGC |
| ALYREF QPCR R | AGAGTTCCTGAATATCGGCG |
| C1D QPCR F | GTTGCAGAAGTTGGATCCAC |
| C1D QPCR R | GCCAGCCTTTTTCTTGTCTG |
| GAPDH QPCR F | GTCTCCTCTGACTTCAACAGCG |
| GAPDH QPCR R | ACCACCCTGTTGCTGTAGCCAA |
| MTR4 QPCR F | GGAGAGATGCAGGTTGTCCCAG |
| MTR4 QPCR R | CTCTGTCTATTGTCCACCGGCC |
| PABPN1 QPCR F | TGTTGGCAATGTGGACTATG |
| PABPN1 QPCR R | CACACAGTATGGTAACACGG |
| PAPD5 QPCR F | CAGCGCTGACGTCCAGATAT |
| PAPD5 QPCR R | CCTCATCTGCGACTTTGTGT |
| RBM7 QPCR F | ACAAGCAGTGATGAACAGTG |
| RBM7 QPCR R | GCTTGAAGACTGATTGAAAC |
| THOC2 QPCR F | GTAGAGAAACCACCTGATAACC |
| THOC2 QPCR R | GCTTGTGTGAAGCTGCATAG |
| UAP56 QPCR F | TCCGTCAGAAGTCCAGCATGA |
| UAP56 QPCR R | ACCAGTACAGACACCTGCCCAGTAA |
| ZCCHC7 QPCR F | CAGATAATGTTTTTGTTGGC |
| ZCCHC7 QPCR R | GTTACTGAAGGAGAAGATGG |
| ZCCHC8 QPCR F | CTCGACCGAGTGGAATATTG |
| ZCCHC8 QPCR R | CTTGATGATATTGCTTTGAAATAGC |
| ZFC3H1 QPCR F | AGTCCTACAGATTTAGTCCA |
| ZFC3H1 QPCR R | GTATATGCTGCCATTGACAAT |
