# Supplementary material for: Nucleolar Proteomics Revealed the Regulation of RNA Exosome Localization by MTR4
Source: Mol Cell Proteomics. 2025 Jul 10;24(8):101031. doi: 10.1016/j.mcpro.2025.101031 (PMC12356310; doi:10.1016/j.mcpro.2025.101031)
Supplement: Table S6 [file mmc6.docx]

**Table S6:** **Antibodies used for Western blots and immunofluorescence.**

| **Antibodies** | **Catalog No.** | **Manufacturer's Name** |
| --- | --- | --- |
| CUGBP1 (CELF1) | ab9549 | Abcam |
| DDX21 | 66925-1-lg | Proteintech |
| EXOSC1 | ab181108 | Thermo Fisher Scientific |
| EXOSC10 | ab50558 | Abcam |
| EXOSC5 | PA5-57927 | Thermo Fisher Scientific |
| Fibrillarin | ab5821 | Abcam |
| LaminB1 | ab16048 | Abcam |
| MTR4 | ab70551 | Abcam |
| MTR4 | PA557927 | Thermo Fisher Scientific |
| NPM1 (B23) | FC- 61991 | Thermo Fisher Scientific |
| PTBP2 | ab154787 | Abcam |
| UBF | sc-13125 | Santa Cruz Biotechnology |
| β-actin | AF5003 | Beyotime |
| Alexa Fluor 488 goat-anti-mouse | A11001 | Thermo Fisher Scientific |
| Alexa Fluor 555 goat-anti-rabbit | A21428 | Thermo Fisher Scientific |
| Goat anti-Mouse | A0216 | Beyotime |
| Goat anti-Rabbit | ab205718 | Abcam |
