# Supplementary material for: Nucleolar Proteomics Revealed the Regulation of RNA Exosome Localization by MTR4
Source: Mol Cell Proteomics. 2025 Jul 10;24(8):101031. doi: 10.1016/j.mcpro.2025.101031 (PMC12356310; doi:10.1016/j.mcpro.2025.101031)
Supplement: Supplemental Figures [file mmc7.pdf]

Supplementary figure legends

Figure S1. Verification of the nucleolus purity for mass spectrometry.

(A)(B) HeLa cells were fractionated into cytoplasmic, nuclear, nucleoplasmic, and nucleolar fractions, followed by western blotting to examine the purity of the nucleolar fractions. β-actin was used as a marker for cytoplasmic fractions, Fibrillarin for nucleolar fractions, and LaminB1 for nucleoplasmic fractions. (C) Quantitative analysis of the nucleolar marker proteins and mitochondrial proteins within the nucleolar proteome dataset, based on three independent biological replicates of control cell nucleoli. (D) The Venn diagrams compared proteins identified in this study with nucleolar proteins reported in a previous study [1], highlighting consistently observed proteins. (E) Nucleolar proteome obtained from HeLa cells following 72 hours RNAi targeting MTR4 were analyzed using the MA plot.

Figure S2. GO analysis of the nucleolar proteome after MTR4 knockdown.

(A) Differential protein accumulation in the nucleoli of MTR4-KD HeLa cells was analyzed using Gene Ontology (GO) enrichment assays. The results were annotated and categorized into different groups based on associated biological processes, cellular components, and molecular functions. (B) List of upregulated nucleolar proteins after MTR4 RNAi across three independent experiments. (C) Relative protein levels of the EXOSC1 and EXOSC5 in control and MTR4-depleted cells within the nucleolar proteome dataset.

Figure S3. PTBP2 and CELF1 were enriched in nucleoli upon MTR4 knockdown.

(A)(D) Images showed the localization of PTBP2 and CELF1 in HeLa(EGFP-EXOSC10) cells following MTR4 knockdown by RNAi. A non-targeting siRNA was used as a control. (B)(E) Western blot analysis of PTBP2 and CELF1 in nucleolar fractions after MTR4 knockdown. The levels of these proteins in the nucleolus were quantified by ImageJ. (C)(F) Western blot analysis of PTBP2 and CELF1 in whole cell lysates after MTR4 knockdown. The levels of these proteins in whole cells were quantified by ImageJ.

Figure S4. The depletion of MTR4 did not influence nucleolar mobility.

(A) HeLa cells were immunostained with EXOSC10 antibody following 72 hours of RNAi targeting MTR4. The white arrows indicate EXOSC10. The white lines indicate the area where fluorescence quantification was performed. (B) Western blot analysis of EGFP-EXOSC10 following MTR4 knockdown by siRNA transfection for 72 hours. A non-targeting siRNA was used as a control. (C) The efficiency of EXOSC10 knockdown was analyzed by western blotting with the indicated antibodies. (D) Images showed that the depletion of EXOSC10 by RNAi did not noticeably impact the nucleolar accumulation of EGFP-MTR4 in HeLa cells. (E-F) (Top) HeLa cells expressing mCherry-B23 or FBL-mCherry were analyzed using FRAP in the indicated regions. (Bottom) Quantification of mCherry-B23 or FBL-mCherry mobility is shown as mean ± s.d. (n=3). The first 90 seconds (E) and 120 seconds (F) after photobleaching were displayed.

Figure S5. The subunits of the RNA exosome complex influence each other's localization.

(A) The percentage of relative EXOSC1 distribution in the nucleolus and nucleoplasm following 72 hours RNAi targeting MTR4. The data were calculated from independent microscopic images (n = 3; total cell number: 500–700). (B) The percentage of relative EXOSC5 distribution in the nucleolus and nucleoplasm following 72 hours RNAi targeting MTR4. The data were calculated from independent microscopic images (n = 3; total cell number: 500–700). (C) The percentage of relative EXOSC5 distribution in the nucleolus and nucleoplasm following 72 hours RNAi targeting EXOSC1 or EXOSC10. The data were calculated from independent microscopic images (n = 3; total cell number: 500–700). (D) The percentage of relative EXOSC10 distribution in the nucleolus and nucleoplasm following 72 hours RNAi targeting EXOSC1 or EXOSC5. The data were calculated from independent microscopic images (n = 3; total cell number: 500–700). (E) Western blot analysis was performed to assess the protein levels of EXOSC1 and EXOSC5 following MTR4 or EXOSC10 knockdown.

Figure S6. MTR4 specifically regulates the nucleolar accumulation of the RNA exosome.

(A)(D) Fluorescent images showed the subcellular localization of EGFP-EXOSC10 in HeLa cells after transfection with siRNAs targeting components of the PAXT, NEXT, and TREX complexes. (B)(E) RT-qPCR analysis was performed to assess the knockdown efficiency of PAXT, NEXT, and TREX complexes in siRNA-treated HeLa cells. Data are presented as mean ± s.d.; n= 3. (C)(F) The efficiency of PAXT, NEXT, and TREX complex components depletion was analyzed using western blotting with the indicated antibodies.

Figure S7. Conditions that did not deplete nucleolar accumulation of EXOSC10.

(A) FCCP and (B) CHX treatments or (C) serum starvation did not induce the depletion of EGFP-EXOSC10 from the nucleolus.

Figure S8. Act.D treatment depletes RNA exosome components from the nucleolus by modulating MTR4.

(A) MA plot identified differential proteins in nucleolar proteome following Act.D treatment. (B) The Venn diagrams showed differentially regulated proteins across two independent experiments. Fold changes higher than 1.43-fold are considered up-regulated, and less than 0.7-fold are considered down-regulated. (C) A list of downregulated proteins following Act.D treatment. (D) The Venn diagrams showed that no proteins were commonly upregulated in both MTR4 knockdown (KD) and Act.D-treated nucleoli. (E) Relative protein levels of the RNA exosome subunits in Act.D-treated cells within the nucleolar proteome dataset.
